# Supplementary material for: Modulation of occluding junctions alters the hematopoietic niche to trigger immune activation
Source: eLife. 2017 Aug 25;6:e28081. doi: 10.7554/eLife.28081 (PMC5597334; doi:10.7554/eLife.28081)
Supplement: Transparent reporting form [file elife-28081-transrepform.doc]

***eLife’s*transparent reporting form**

We encourage authors to provide detailed information *within their submission* to facilitate the interpretation and replication of experiments. If you have any questions, please contact us:[editorial@elifesciences.org](mailto:editorial@elifesciences.org).

**Sample-size estimation**

- You should state whether an appropriate sample size was computed when the study was being designed
- You shouldstate the statistical method of sample size computation and any required assumptions
- If no explicit power analysis was used, you should describe how you decidedwhat sample (replicate) size (number) to use

Please outline where this information can be found within the submission (e.g., page numbers or figure legends), or explain why this information doesn’t apply to your submission:

Information about Sample-size estimation can be found in “Statistical analysis and significance” sub-section in the Materials and Methods section on Pages 43-44 of the main text.

**Replicates**

- You should report how often each experiment was performed
- You should includea definition of biological versus technical replication
- The data obtained should be provided and sufficient information should be provided to indicate the number of independent biological and/or technical replicates
- If you encountered any outliers, you should describe how these were handled
- Criteria for exclusion/inclusion of data should be clearly stated
- High-throughput sequence data should be uploaded before submission, with a private link for reviewers provided (these are available from both GEO and ArrayExpress)

Please outline where this information can be found within the submission (e.g., page numbers or figure legends), or explain why this information doesn’t apply to your submission:

1) Information about replicates can be found in “Statistical analysis and significance” sub-section in the Materials and Methods section on Pages 43-44 of the main text.
2) Information about replicates on survival assays upon oral/systemic infection can be found in the materials and methods section on Pages 41-42.

**Statistical reporting**

- Statistical analysis methods should be described and justified
- Raw data should be presented in figures whenever informative to do so (typically when N per group is less than 10)
- For each experiment,you should identify the statistical tests used, exact values of N, definitions of center, methods of multiple test correction, and dispersion and precision measures (e.g., mean, median, SD, SEM, confidence intervals; and, for the major substantive results, a measure of effect size (e.g., Pearson's r, Cohen's d)
- Report exact p-values wherever possible alongside the summary statistics and 95% confidence intervals. These should be reported for all key questions and not only when the p-value is less than 0.05.

Please outline where this information can be found within the submission (e.g., page numbers or figure legends), or explain why this information doesn’t apply to your submission:

1) Information about statistical reporting can be found in the figure legends for the Main figures and the associated figure supplements for the main figures on Pages 25-36.
2) This information is also stated in a detailed manner in the “Statistical analysis and significance” sub-section of the Materials and methods section on Pages 43-44 in the main text.

(For large datasets, or papers with a very large number of statistical tests, you may upload a single table file with tests, Ns, etc., with reference to page numbers in the manuscript.)

**Additional data files (“source data”)**

- We encourage you to upload relevant additional data files, such asnumerical data that are represented as a graph in a figure, or as a summary table
- Where provided, these should be in the most useful format, and they can be uploaded as “Source data” files linked to a main figureor table
- Include model definition files including the full list of parameters used
- Include code used for data analysis (e.g., R, MatLab)
- Avoid statingthat data files are “available upon request”

Please indicate the figures or tables for which source data files have been provided:

Multiple source data files have been uploaded for Figures 1-5, 7 and 9.
